# Differential Physical and Mental Benefits of Physiotherapy Program Among Patients With Schizophrenia and Healthy Controls Suggesting Different Physical Characteristics and Needs

**DOI:** 10.3389/fpsyt.2021.536767

**Published:** 2021-02-09

**Authors:** Michele Fonseca Szortyka, Viviane Batista Cristiano, Paulo Belmonte-de-Abreu

**Affiliations:** ^1^Graduate Program in Psychiatry and Behavioral Sciences, Federal University of Rio Grande Do Sul, Porto Alegre, Brazil; ^2^Schizophrenia Program of Hospital de Clínicas de Porto Alegre, Porto Alegre, Brazil; ^3^Department of Psychiatry, Schizophrenia Program of the Federal University of Rio Grande Do Sul Medical School, Hospital de Clínicas de Porto Alegre, Porto Alegre, Brazil

**Keywords:** exercise, physical activity, 6-min walk test, functional capacity, schizophrenia

## Abstract

In contrast to several other severe illnesses marked by inflammation and autoimmunity that now have potent and efficient treatments and even cures, schizophrenia (SCZ) is a disease still associated with poor outcome, incapacity, and social burden. Even after decades of research on the brain and behavior, this illness is still associated with profound effects on both mental health and physical health, with recent studies showing that treatment is more efficient when associating drugs with psychological and physical treatments. Most of the studies measured the effects of physical intervention compared with usual care and demonstrated a positive effect as an add-on treatment. What remains unclear is the different effects of the same intervention in normal subjects in a sample of patients with the illness. The study aimed to evaluate the effects of physical intervention over motor functional capacity and mental health in patients with SCZ compared with healthy controls (HC). The outcomes were (a) functional capacity [by 6-min walk test (6MWT)], (b) body flexibility index (Wells' bench), (c) disease severity [by Brief Psychiatric Rating Scale (BPRS)], (d) quality of life [by 36-Item Short Form (SF-36) questionnaire], and (e) physical activity [Simple Physical Activity Questionnaire (SIMPAQ)]. The intervention was associated with significant decrease of body mass index (BMI), blood pressure, disease severity, and improvement in daily life activities. Unexpectedly, it was observed that schizophrenics, compared with matched HC, were at a lower level of performance in the beginning, remained below HC over the studied time despite similar physical intervention, and had different changes. The intervention had lower effects over physical capacity and better effects over quality of life and disease severity. The results confirm previous studies comparing patients receiving physical intervention but suggest that they may receive different types of intervention, suited for their different baseline fitness, motivation, and capacity to engage in physical effort over sustained time. Additionally, they point to extended time of intervention of multidisciplinary treatment (physical and psychological–cognitive techniques) to improve outcomes in SCZ.

## Introduction

Schizophrenia (SCZ) is a leading cause of disability worldwide (1, 2) and affects about 21 million people (3). Patients have three major symptom dimensions, which can be described as positive symptoms (hallucinations and delusions), negative symptoms (affective flattening, alogia, and avolition), and cognitive symptoms (perception, memory, and attention) (4, 5). Additionally, these patients are 1.5–2 times more likely to be overweight, with a two-fold risk for diabetes and hypertension and a five times risk for dyslipidemia than the general population (3, 6, 7).

The association of SCZ with cardiometabolic risk factors reveals a complex interplay between environmental physical inactivity, unhealthy diet, substance abuse, genetics and illness-related factors, and effects of antipsychotic treatment (6, 8). Previous studies also reported association between low functional exercise capacity and low level of perceived sports competence, perceived physical fitness, and physical activity (PA) involvement (9, 10).

PA can be defined by bodily movement produced by skeletal muscles requiring energy expenditure (11). Therefore, only a minority (about 25%) of patients with SCZ do not meet the minimum public health recommendation of 150 min of moderate–vigorous PA per week (6, 12). As evidenced by previous studies, reduced PA substantially affects quality of life, functionality, and physical health (11, 13).

Reduced PA in patients with SCZ is related to disease severity, symptoms, illness durations, types of antipsychotic drugs, and intensity of extrapyramidal side effects (12). Additionally, patients have low motivation and dislike regular exhausting exercises (14). The consequences extend to the brain level, since exercise induces neurogenesis, modulates synaptic plasticity, and increases several growth factors relevant for brain function (12, 15).

The 6-min walk test (6MWT) has been studied in patients with SCZ and considered an adequate submaximal test for these patients (2). It is a self-paced test, better tolerated and more reflective of daily activity than other maximal exercise tests. It is practical, simple, and easy to perform with minimal equipment (6, 10) and provides information regarding the functional exercise capacity of individuals by measuring the distance that they can quickly walk on a flat, hard surface in a period of 6 min (10, 16).

This study aimed to evaluate the functional capacity (6MWT), physical inactivity [Simple Physical Activity Questionnaire (SIMPAQ)], symptom severity [Brief Psychiatric Rating Scale (BPRS)], and quality of life [36-Item Short Form (SF-36)] and to observe the effects of an aerobic intervention in two groups: those with an SCZ diagnosis and matched healthy controls (HC) (paired by sex, age, and social class).

## Materials and Methods

### Trial Design

This is a paired clinical trial of physical intervention [aerobic physical intervention program (APIP)] in a group of stable outpatients with a diagnosis of SCZ. All patients received regular care at a public health facility [Center of Psychosocial Attention (CAPS)] in a mid-sized town of southern Brazil (Camaquã) and were compared with HC paired by sex, age, and social class.

### Participants

Stable outpatients under regular treatment at the CAPS, in Camaquã, state of Rio Grande do Sul, Brazil, received a psychiatric diagnosis after a three-step procedure consisting of (1) careful clinical observation with at least three evaluations; (2) a family interview; and (3) a review of their medical records performed by a trained psychiatrist. The selected patients met the following inclusion criteria: *Diagnostic and Statistical Manual of Mental Disorders*, 5th Edition (DSM-5) (17) and *International Classification of Diseases*, Tenth Revision (ICD-10) diagnoses of SCZ; were aged between 18 and 65 years; were under stable drug treatment adjusted to their clinical state for at least 3 months; and were not involved in other PA programs during the intervention. Exclusion criteria were alcohol or other drug abuse in the last month; major systemic or neurological diseases; physical disability contraindicating PA; risk of suicide confirmed by direct contact with the patient and family; pregnancy or women of reproductive age who did not use a contraception method; and not agreeing to participate in the study after full explanation of the program. The sample size was calculated using the WINPEPI program (version 10.5), with estimated sample size of 30 patients with a diagnosis of SCZ and 30 HC.

The paired HC were recruited through specific social networks with the following inclusion criteria: same sex as the patient in question; similar age (3 years older or younger); same social class; and absence of any major mental illness defined by enrollment interview using direct questioning of lifetime experiences of memory loss, psychosis (delusions and or hallucinations), depression, mania, generalized anxiety disorder, or obsessive–compulsive disorder. Exclusion criteria were the same as those applied for patients with SCZ.

### Ethics

The study was registered in the Brazilian Research Registry under No. 43408615.7.0000.5327, registered in the Brazilian Registry of Clinical Trials (ReBEC) under No. RBR-2h2hjy, and approved (150066) by the Research Ethics Committee of *Hospital de Cl*í*nicas de Porto Alegre* (HCPA). Patients and their legal guardians provided written informed consent after reading and understanding the intervention program and their rights.

### Clinical Assessment

After patient recruitment, previously trained professionals performed standardized clinical and physical assessment of the study participants before physical intervention and after 3 months of treatment.

### Disease Severity: Brief Psychiatric Rating Scale

The BPRS is one of the most widely used instruments to evaluate the presence and severity of various psychiatric symptoms; it is currently used by the Brazilian Unified Health System (SUS) for patient monitoring (18). This tool assesses 18 domains of symptoms: somatic concern, anxiety, emotional withdrawal, conceptual disorganization, guilt feelings, tension, mannerisms and posturing, grandiosity, depressive mood, hostility, suspiciousness, hallucinatory behavior, motor retardation, uncooperativeness, unusual thought content, blunted affect, excitement, and disorientation. The assessment takes ~5–10 min, following an interview with the patient, and the clinician rates each item on a scale ranging from 0 (not present) to 6 (extremely severe) through observation and questioning depending on the assessed item.

### Physical Performance: The 6-Min Walk Test

The 6MWT was applied by two trained and certified physical therapists according to the American Thoracic Society Guidelines (2002) (19). The test was performed in a corridor containing minimal external stimuli and demarcated turnaround points. The participants received careful instructions to walk as briskly as possible, without running, during 6 min; they should do their best during this time but could stop if needed. The technicians used standard encouragement words and attitudes throughout the test. Blood pressure (BP), heart rate, respiratory rate, peripheral blood oxygen saturation, and dyspnea (measured by Borg's perceived exertion scale) were measured at the beginning, in the third minute, and at the end of the test. Algorithms by Enright and Sherrill (2003) (20) predicted ranges for 6-min walk distances (6MWD) using sex, height, age, and weight parameters.

### Stretching: Wells' Bench

The Wells' sit-and-reach flexibility test was used for measuring the flexibility of the posterior muscles of the lower limbs and the mobility of the hip joint. The participant sat on the floor or exercise mat with fully extended legs and soles of the feet against the bench and slowly bent over and projected forward as far as possible, with the fingers sliding along a scale. The total distance reached after three attempts provided the final score (21).

### Quality of Life: 36-Item Short Form

The Medical Outcomes Study SF-36 is a commonly used, validated questionnaire method with high sensitivity to detect functional status, among other aspects of quality of life. The SF-36 questionnaire includes eight multiple-item subscales that evaluate functional capacity, physical limitation, pain, general health, vitality, social aspects, emotional limitations, and mental health. The total score on each SF-36 subscale ranges between 0 and 100; the higher the score, the better the patient is.

### Physical Activity: Simple Physical Activity Questionnaire

The SIMPAQ is a five-item clinical tool designed to assess PA among populations at high risk of sedentary behavior. The PA questionnaire evaluates the last 7 days including time in bed, sedentary time, time spent walking, type and time spent exercising, and time spent in other activities, including leisure, domestic, work, and transportation activities.

### Physical Intervention: Aerobic Physical Intervention Program

The patients continued receiving regular clinical treatment in addition to the standardized APIP. This 12-week program included 1-h sessions of aerobic exercise twice a week. Participants were monitored during the exercises by a digital rate monitor (POLAR FT1®, USA); and their results were adjusted by age, sex, weight, and height. Our measurements ranged from 70 to 80% of maximum heart rates calculated by the Karvonen formula (22). A standard session began with a 5-min warm-up of comfortable intensity and continued with an aerobic exercise of increasing intensity using any of the three modalities: a stationary bicycle (Embreex 367C, Brazil), a treadmill (Embreex 566BX, Brazil), or an elliptical trainer (Embreex 219, Brazil). This strategy was consistent with public health recommendations that suggest an adaptation of the program to individual preferences and has demonstrated feasibility in patients with a diagnosis of SCZ (23, 24). A trained professional coordinated the intervention sessions with guidance, adjustments of the equipment, and encouragement of the participant's exercise performance as best as possible for each patient. After completing aerobic exercise, participants performed global stretching of large muscle groups. Heart monitors recorded initial heart rates, maximum heart rates, and calories expended during a session. Calories were calculated within a range of 7–16 kcal/kg. For example, if the patient was 60 kg, he would need to spend between 420 and 960 kcal per week with APIP. In both groups (patients with SCZ and HC), there could not be more than 10% absent in the physical intervention for their data to be included in the research.

### Statistical Analyses

Data in Excel® were exported to SPSS® v20.0. Categorical variables were described by frequency and percentage. Kolmogorov–Smirnov test assessed data symmetry. Quantitative variables with symmetrical distribution were described by the mean and standard deviation and those with asymmetric distribution by the median and interquartile range. Categorical variables were compared using the McNemar test. Quantitative variables with symmetrical distribution were compared using Student's *t*-test for paired samples. The variables with asymmetric distribution were compared using the Wilcoxon test. To assess the correlation between quantitative variables, Pearson's or Spearman's correlation coefficient was used. The defined significance level was 5%.

## Results

**Figure 1:**
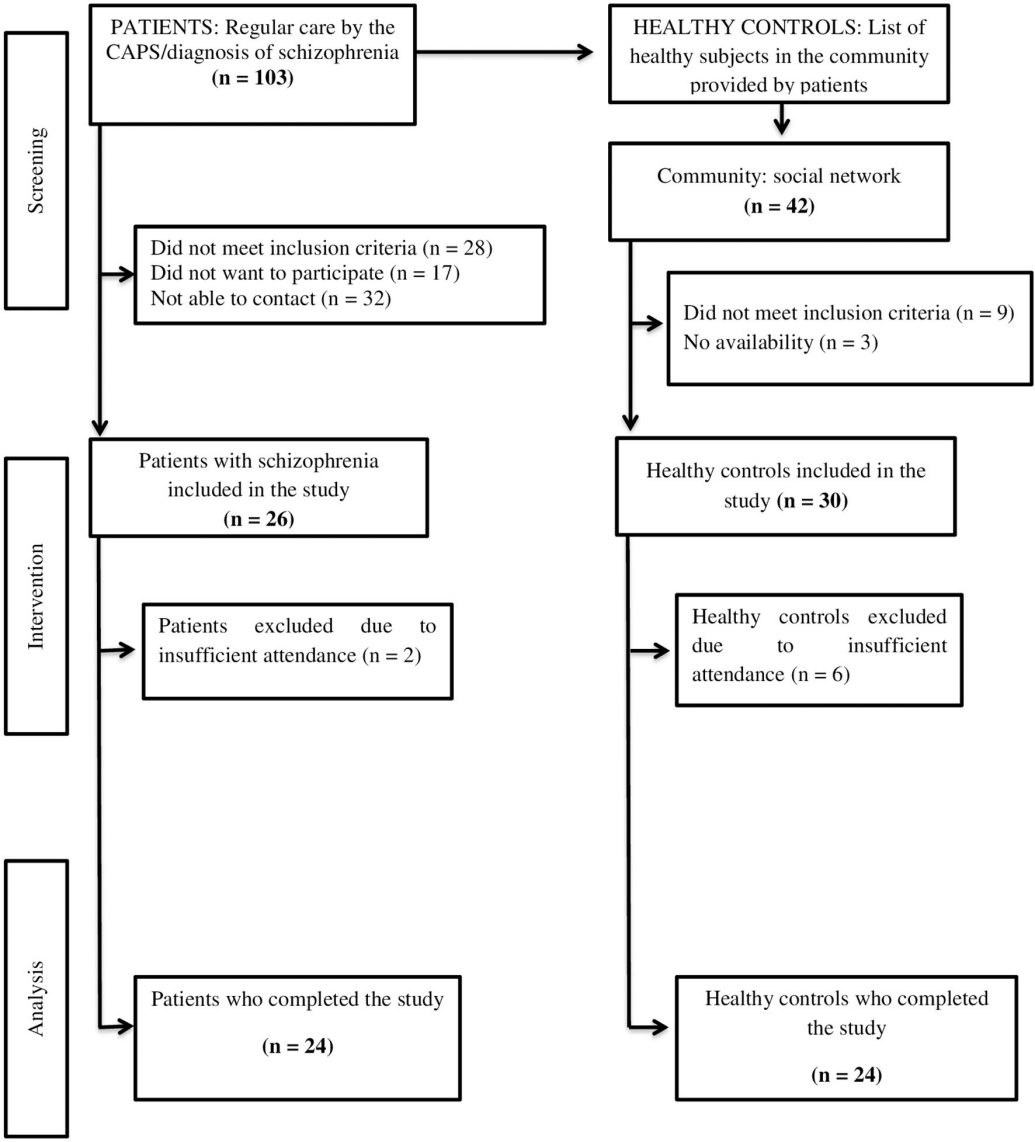
Study flowchart: Aerobic physical intervention program in patients with schizophrenia and healthy controls.

Out of the 103 patients with the diagnosis of SCZ that were initially invited, 26 agreed to participate and met the inclusion criteria. Of these, 24 patients (92%) completed the physical exercise intervention. Dropouts consisted of two patients (8%) due to not having a minimum frequency of 90% in APIP. A total of 24 from the original 30 (80%) paired HC completed the APIP, and six patients (20%) were excluded due to not having the minimum frequency (Figure 1).

The sample of patients with the diagnosis of SCZ (Table 1) included males (83.3%), with a mean 40.75 years of age (±SD 12.20 years) and weighing 90.50 (±SD 2.48) kg, and the mean height was 1.68 (±SD 0.06) m. After intervention, patients with SCZ lost a mean body weight of 1.98 kg (±SD 3.50 kg) (*p* = 0.011), and controls 1.01 kg (±SD 2.42 kg) (*p* = 0.053). Body mass index (BMI) was significantly reduced in the cases (*p* = 0.002) and had a trend to be reduced in HC (*p* = 0.088). Systolic and diastolic BP was significantly reduced in the patients with SCZ and HC (*p* = 0.017 and *p* = 0.003, respectively); and flexibility changed from baseline 16 cm in cases and 15 cm in HC, to 17 cm (*p* = 0.299, non-significant) and 17 cm (*p* < 0.001), respectively. Table 2 shows the results pre and post the intervention of cases and controls on the BPRS, SIMPAQ, and SF-36 scale. The BPRS scale was applied only in the cases because it is a scale of severity of various psychiatric symptoms.

**Table 1:** Comparative table of characteristics of included subjects.

**Characteristics**	**Cases *N* (24)**	**Controls *N* (24)**	***p***
Gender male, *n* (%)	20 (83.3)	20 (83.3)	1.000^*^
Basic education, *n* (%)	24 (100.0)	20 (83.3)	0.999^*^
Single marital status, *n* (%)	23 (95.8)	6 (25.0)	<0.001^*^
Smoker, *n* (%)	7 (29.2)	0	0.009^*^
Weight, mean ± SD	89.4 ± 24.5	92.3 ± 16.3	0.631^**^
BMI, mean ± SD	31.6 ± 8.4	31.1 ± 5.5	0.828^**^
Systolic BP, mean ± SD	125.0 ± 10.6	125.4 ± 14.1	0.914^**^
Diastolic BP, mean ± SD	82.9 ± 10.8	82.9 ± 12.7	0.999^**^
Flexibility, mean ± SD	16.4 ± 8.6	15.0 ± 8.7	0.574^**^

*SD, standard deviation; BMI, body mass index; BP, blood pressure*.

** *McNemar test*.

* *Paired t-test*.

**Table 2:** Comparison of pre and post in physical and mental variables measured during 3 months of APIP in patients with schizophrenia.

**Variables**	Cases^*^N **(24)**		**p cases**	Controls^*^N **(24)**		**p controls**
BPRS^**^	**Pre**	**Post**		**Pre**	**Post**	
Somatic preoccupation	1.21 ± 1.53	1.08 ± 1.38	0.808	–	–	–
Anxiety	1.58 ± 1.58	1.66 ± 1.94	0.875	–	–	–
Affective withdraw	1.08 ± 1.69	1.45 ± 1.71	0.202	–	–	–
Conceptual disorganization	1.92 ± 2.10	1.38 ± 1.86	0.101	–	–	–
Guilt	1.29 ± 1.73	1.25 ± 1.53	0.874	–	–	–
Tension	0.25 ± 0.84	0.5 ± 1.38	0.596	–	–	–
Mannerism	0.17 ± 0.56	0.66 ± 1.43	0.066	–	–	–
Grandiosity	0.56 ± 1.28	0.5 ± 1.14	0.680	–	–	–
Depressive mood	1.67 ± 2.35	1.83 ± 2.14	0.658	–	–	–
Hostility	0.67 ± 1.09	1.21 ± 1.88	0.138	–	–	–
Paranoid ideation	1.04 ± 1.78	1.54 ± 1.91	0.163	–	–	–
Hallucination	0.96 ± 1.54	1.20 ± 1.79	0.480	–	–	–
Psychomotor retardation	1.33 ± 1.99	1.45 ± 1.79	0.885	–	–	–
Lack of cooperation	0.17 ± 0.63	0.58 ± 1.31	0.118	–	–	–
Delusions	1.13 ± 1.65	1.46 ± 1.93	0.550	–	–	–
Affect	1.92 ± 2.08	1.71 ± 1.85	0.700	–	–	–
Excitement	0.13 ± 0.44	0.37 ± 0.92	0.245	–	–	–
Disorientation	1.25 ± 1.48	0.75 ± 1.39	0.184	–	–	–
SIMPAQ^**^						
Time in bed	607.5 ± 114	586.2 ± 84.8	0.339	466.2 ± 82.5	442.9 ± 67.0	0.202
Sedentary time	365 ± 144.7	368.7 ± 162.9	0.763	427.7 ± 107.5	337.2 ± 111.7	**0.018**
Time spent walking	161.2 ± 211.8	195.4 ± 271.5	0.129	132.9 ± 150.5	309.8 ± 309.4	**0.003**
Type exercise	16.6 ± 44.3	126.6 ± 140.7	**<0.001**	20.0 ± 70.0	202.5 ± 175.4	**<0.001**
Other activities	34.5 ± 76.4	42.9 ± 75.8	0.733	69.3 ± 107.4	158.5 ± 180.4	**0.047**
SF-36^***^						
Functional capacity	66.5 ± 30.8	80.6 ± 20.3	**0.025**	75.8 ± 16.6	85.2 ± 12.2	**0.002**
Physical limitation	47.8 ± 45.1	57.6 ± 37.2	0.377	68.3 ± 31.8	79.7 ± 27.9	**0.024**
Pain	69.1 ± 26.5	61.6 ± 34.4	0.351	59.6 ± 27.1	74.6 ± 23.8	**0.003**
General health	45.1 ± 23.5	54.8 ± 23.2	0.171	53.4 ± 17.5	61.9 ± 18.3	**0.040**
Vitality	57.7 ± 27.8	66.1 ± 29.3	0.391	62.0 ± 18.8	71.0 ± 17.5	**0.007**
Social aspects	59.7 ± 24.8	66.2 ± 23.4	0.305	73.7 ± 28.0	83.2 ± 22.1	**0.034**
Emotional limitations	42.3 ± 45.0	60.5 ± 44.4	0.130	70.5 ± 30.5	84.3 ± 24.7	**0.024**
Mental health	65.6 ± 25.5	66 ± 26.8	0.936	73.9 ± 20.7	81.6 ± 19.5	**0.037**

*BPRS, Brief Psychiatric Rating Scale; SIMPAQ, Simple Physical Activity Questionnaire; SF-36, The Medical Outcomes Study 36-Item Short Form; APIP, aerobic physical intervention program*.

* *Date is presented as mean ± standard deviation; p < 0.05*.

** *Wilcoxon test for variables with asymmetric distribution*.

*** *Paired t-test for samples with normal distribution. The values in bold that are marked in the variables are the results of the p < 0.05*.

### 6-Min Walk Test

**Figure 2:**
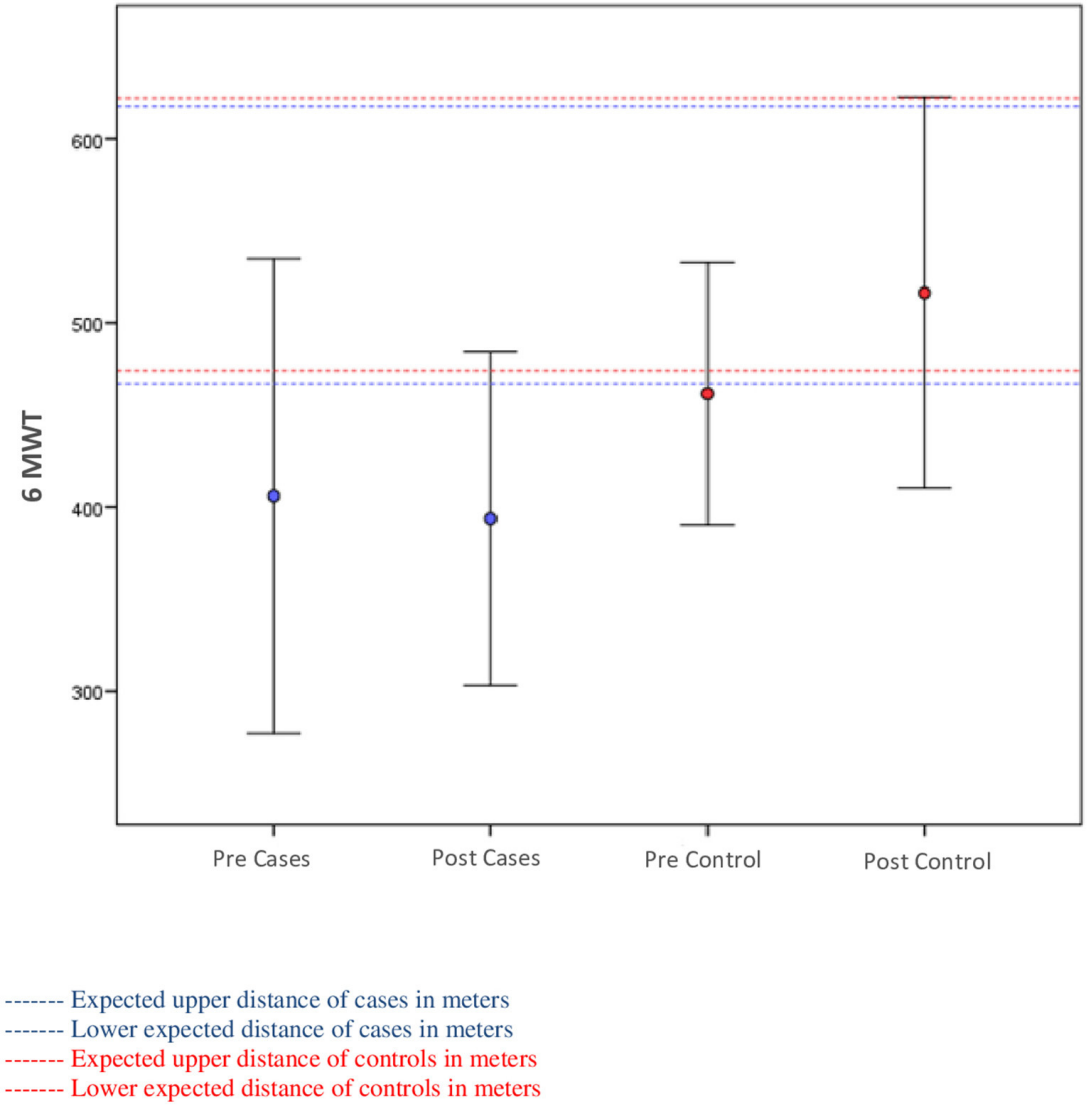
Relationship between the distances walked on the 6-min walk test (6MWT) and among the predicted according to the Enright et al. equation of cases and controls.

Figure 2 displays 6MWT changes. Cases walked initially 406 m (±SD 128 m) and HC 461 m (±SD 71 m). Patients with SCZ were 13% below the predicted minimum, and even after APIP, they remained 15% below predicted. Patients with SCZ failed to improve 6MWT (*p* = 0.531), whereas HC had significant improvement (*p* = 0.015). Additionally, the mean change of patients with SCZ was significantly different from that of HC (*p* = 0.022).

### Brief Psychiatric Rating Scale

Table 3 describes BPRS changes with 6MWT changes. Functional capacity was associated with reduction of manic symptoms (*r* = 0.47, *p* = 0.021), reduction in disorientation (*r* = 0.42, *p* = 0.039), and improvement in cooperation with the interviewer (*r* = 0.47, *p* = 0.022).

**Table 3:** Correlation changes 6MWT and BPRS.

**BPRS change**	**6MWT_PP change**	
	***r***	***p***
1- Somatic preoccupation	0.08	0.703
2- Anxiety	0.30	0.160
3- Affective withdraw	−0.24	0.251
4- Conceptual disorganization	0.13	0.545
5- Guilt	0.22	0.310
6- Tension	0.29	0.172
7- Mannerism	0.47	0.021
8- Grandiosity	−0.60	0.002
9- Depressive Mood	−0.22	0.311
10- Hostility	0.03	0.890
11- Paranoid Ideation	0.03	0.890
12- Hallucination	0.08	0.697
13- Psychomotor Retardation	0.20	0.356
14- Lack of cooperation	0.47	0.022
15- Delusions	0.04	0.841
16- Affect	−0.23	0.281
17- Excitement	−0.05	0.815
18- Disorientation	0.42	0.039
1- Anxiety/Depression (5,2,9,1)	0.10	0.645
2- Retardation (13,16,3,18,14,7)	0.08	0.714
3- Thought Dis 11,15,12,4,10,8	−0.20	0.355
4- Activation 7,6,17,8	0.07	0.742
5- Hostility 10,11,14,8	−0.06	0.776

*BPRS, Brief Psychiatric Rating Scale; 6MWT PP, 6 min walk test, predictive percentage*.

### 36-Item Short Form Quality of Life Scale

Patients with SCZ increased about 10% in almost all SF-36 domains, but only one (physical functional capacity–daily life activities performance) had significant improvement (*p* = 0.025). In contrast, HC improved significantly in all SF-36 domains [functional capacity (*p* = 0.002), physical limitation (*p* = 0.024), pain (*p* = 0.003), general health (*p* = 0.040), vitality (*p* = 0.007), social aspects (*p* = 0.034), emotional limitations (*p* = 0.024), and mental health (*p* = 0.037)]. HC also had significantly higher improvement in pain (intensity and interference in daily activities) (*p* = 0.031) than had patients with SCZ.

### Simple Physical Activity Questionnaire

The SCZ group had a significant change in time spent exercising from 16 to 126 min per week (*p* < 0.001) without changes in the remaining SIMPAQ items of daily sleep time (from 607 to 586 min/day; *p* = 0.339); daily sedentary time (from 365 to 368 min/day; *p* = 0.764); weekly walking time (from 165 to 195 min/week; *p* = 0.129); and weekly time on other activities (from 34 to 42 min/week; *p* = 0.733). In contrast, HC had improvement in four measures: (a) sedentary time (from 427 to 337 min/day; *p* = 0.018), (b) time walking (from 132 to 309 min/week; *p* = 0.004), (c) time on physical exercise (from 20 to 202 min/week; *p* < 0.001), and (d) time on other activities (from 69 to 158 min/week; *p* = 0.048). Sleeping time in HC remained stable (from 466 to 442 min/day, *p* = 0.202).

### Weekly Calorie Burning

**Figure 3:**
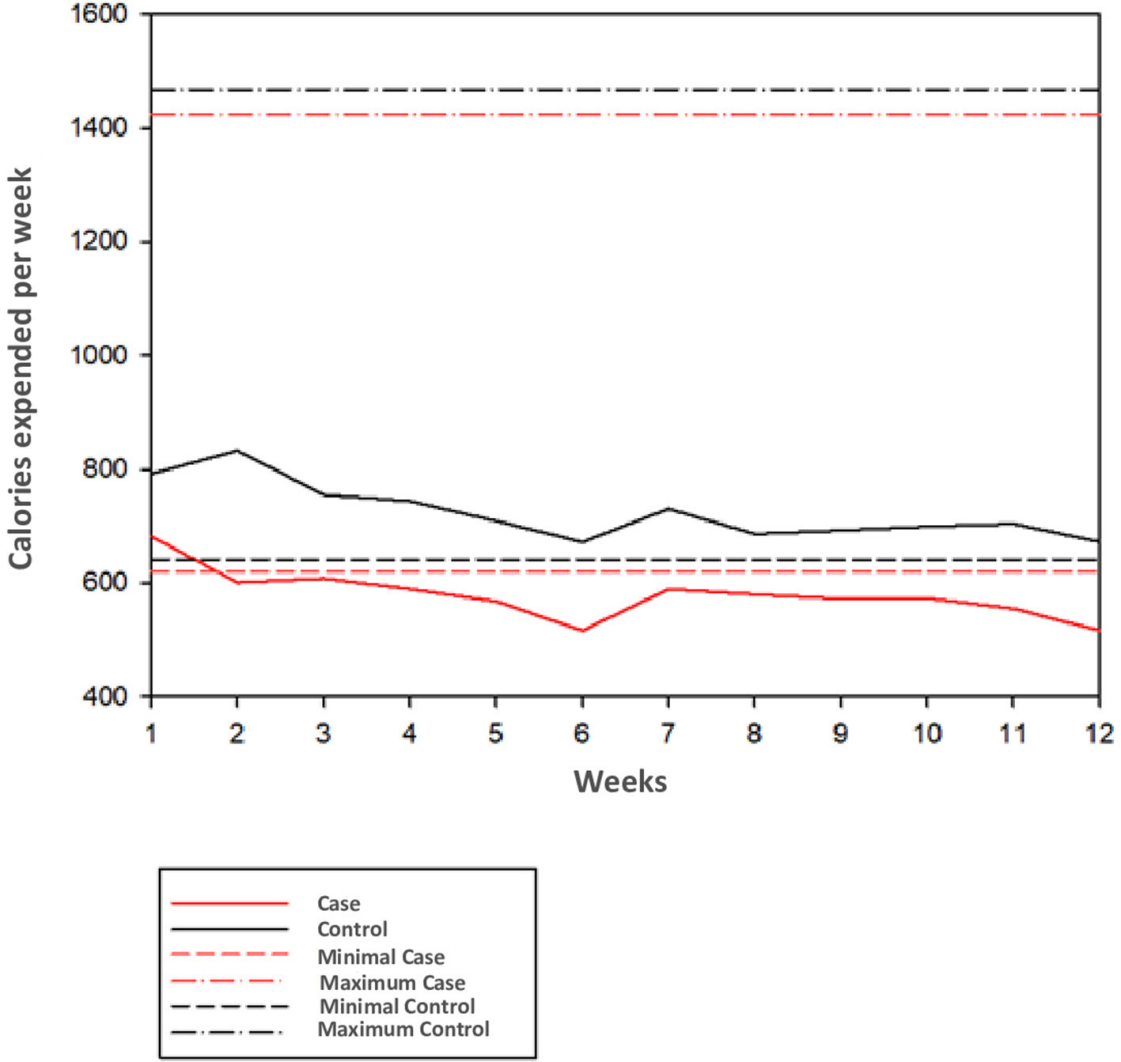
Relationship between calories spent per week and a minimum a maximum expected.

Figure 3 presents a graph showing weekly observed and expected calorie burning in patients with SCZ and HC. Patients with SCZ remained close to the minimum expected in the first 2 weeks and after that remained below expectations. Patients with SCZ initially burned 682 kcal in weeks 1 and 2, decreasing to 516 kcal in the sixth and 12th weeks, representing 16% lower than expected (620 kcal). In contrast, HC caloric burn remained under expected limits from the first to 12th weeks (with 792 kcal in the first week and 672 kcal in the sixth and 12th weeks; 23.5 and 4.8% above inferior expected limit of 641 kcal/week).

## Discussion

The study provided additional evidence of differential effects of physical intervention (APIP) in patients with SCZ and HC, suggesting that patients with SCZ must receive differentiated and individualized approaches in physical treatment. Previous literature highlighted the importance of physical intervention in patients with SCZ and the association of APIP with functional and cognitive improvement. The study focused on the differences of physical intervention in severely mentally ill subjects with the diagnosis of SCZ compared with paired HC and revealed that patients with SCZ have not only different basal capacities but also different performance and thus may receive different APIP than HC. Despite global inferiority of changes in patients with SCZ compared with HC, APIP was associated with significant reduction of BMI and BP, in line with previous studies (2, 8, 10, 25).

The functional capacity is related to the degree of PA, and this relates to physical health and cardiometabolic risk profile. Since about 50% of patients with the diagnosis of SCZ have low levels of PA and consequent increased cardiometabolic risk, increased PA will ultimately reduce those risks (26–28). In addition, a recent meta-analysis with 3,453 patients with SCZ estimated a practice of 80.4 min of light PA, 16.2 min of moderate PA, and only 1.1 min of vigorous PA, with only 56.6% of these subjects performing the recommended 150 min of moderate PA per week (29). Additional systematic reviews estimated 12 sedentary hours/day in patients with SCZ, ~3 h (33%) more than the control group (30). SIMPAQ scale demonstrated that patients with SCZ spent 42% of the day sleeping, spending most of their time being sedentary. The present study, despite failing to demonstrate effects in some areas of activity, was able to remove SCZ patients from a sedentary lifestyle and increase the level of PA. It was observed that a positive correlation of the improvement in the symptomatology of the disease (mannerism, lack of cooperation, and disorientation) occurred when there was an improvement in 6MWT, corroborating with previous studies (4, 10, 12).

Changes in HC are in line with previous studies of Hanson and Jones (24) of 3 and 5% decrease in systolic and diastolic BP, respectively (*p* = 0.001), BMI decrease (*p* = 0.003), and walk increase (6MWT) (*p* = 0.001). In the study, patients with SCZ failed to improve 6MWT performance, despite several other improvements in physical measures (BP and BMI) and symptoms (disorientation and cooperativeness). Additionally, these findings are also in line with Vancampfort et al. (25) results of different performance according to BMI (overweight patients with lower performance than normal weight: 580.2 vs. 615.8 m; *p* < 0.001).

The unexpected finding of increased pain perception in patients with SCZ deserves special considerations, since recent studies demonstrated that patients with SCZ (31–33) have a higher pain threshold for pain than the general population and lower measures of pain than do other patients with major psychiatric disorders, particularly bipolar disorder. As expected (34), patients in early stages of illness had more pain than those with more advanced stages and HC. But pain increase after APIP could reflect even the effects of increased charge over weaker muscles and/or defective posture, especially in the spine, or prior muscle atrophy consequent of low activity. Additionally, the increased report of pain could reflect the unexpected and not necessarily unfavorable outcome of increased body awareness.

The study revealed that the SCZ group had a prevalence of 29% of tobacco use in contrast to 0% in HC, and this was strongly linked to BMI (*p* = 0.003). Since tobacco exposure is an established cause of physical health problems (e.g., cardiovascular disorders, chronic respiratory disorders, lung cancer, and other malignancies), this increased prevalence in patients with SCZ may account for the increase in premature death among individuals with this diagnosis. It also could account for increased levels of psychopathology (general and positive symptoms in BPRS) in the group of SCZ smokers compared with non-smokers (35–37).

The APIP had a stronger effect in HC compared with patients with SCZ, and this could be derived from the failure in providing adequate incentive for patients to perform the exercises with subsequent lower engagement than in HC. Additionally, the difference could reflect different reliability of answers among the groups. Patients with SCZ may provide less reliable answers from deviant behaviors as compared with the observed behaviors. This could be in accordance with Firth et al.'s (14) findings of reduced compliance, activity, energy, disposition, motivation, and support for physical effort. In addition, it is supported by Costa et al.'s (13) findings of low motivation associated with lower cognitive performance, poor functionality, low treatment adherence, and low activity learning in patients with SCZ. Muscular strength can also play a role in the results. Nygård et al. (38) evidenced decreased lower limb strength and accelerated strength loss after the fourth decade in patients with SCZ compared with a healthy population (20% compared with 10% per decade). Although our study failed to assess muscle strength, these findings must be taken into consideration to explain the differences of APIP in motor functional capacity and in PA among patients and HC.

Another important factor could be related to lack of adequate pairing of cases and controls. Although the study subjects were paired by sex, age, and social level, the study failed to pair by basal physical capacity. Schizophrenic patients have intrinsically greater than expected motor functional impairment than subjects with similarity. With this basal difference, it would be expected that patients with SCZ would display a different improvement rhythm that HC. To answer this question, additional studies may select as control group subjects with physically debilitating diseases (metabolic, vascular, or cardiac syndrome) or perhaps controls with the same pathology without performing physical intervention. Additionally, since previous studies showed that high levels of PA are associated with a 27% decrease in the risk of incidence of psychosis–SCZ (11), it may be important to pair the cases with controls with similar PA and general health before illness onset. This is supported by previous studies of Mittal et al. (12) of high risk for psychosis (HRP) associated with lower PA and lower health compared with normal developing adolescents, including increased tobacco use and alcohol abuse, and of Stochl et al. (39) of delayed childhood motor development in HRP.

Another limitation of the study is that it failed to address brain-induced changes with APIP. This is supported by several studies demonstrating positive effects of aerobic PA over the brain level, with hippocampal and white matter volume increase, new cell formation, increased neuronal plasticity, improved gliogenesis, improved synapses, increased astrocyte density, angiogenesis, growth factors [brain-derived neurotrophic factor (BDNF), insulin-like growth factor-1 (IGF-1), and vascular endothelial growth factor (VEGF)] and neurotransmitters like serotonin, norepinephrine, dopamine, glutamate, and acetylcholine (12, 15, 40), all associated with increased memory and cognition. The failure to assess patients at the neuronal level limits further discussion about differential brain effects in patients with SCZ and HC. Anyway, it is possible to point to the possibility of putative effects of PA in patients with SCZ, expressed by the observed outcomes of improved activation and cooperation and reduced hostility and disorientation. Additional study limitations could derive from different rates of regular use of beta-blockers. Since patients with SCZ used it more often than HC, they would have more difficulty in raising the heart rate and consequently to have caloric burn increase with vigorous exercise, although it is not clear if this would reflect a real difference of caloric burn, or a measurement error, since the cardiac monitor calculates caloric expenditure utilizing heart rate, and people under beta-blockers are not able to obtain similar values with similar exercises.

The logistics of the study may have also exerted influence over the different outcomes. When one looks at the graph of expended and expected calories according to the World Health Organization, it can have the idea that it could be indicated to make a two- to three-fold increase in weekly time of exercise. In this study, patients faced access differences to the place of APIP. Patients with SCZ mostly lived in rural areas with underserved public transport and poor road pavement conditions, and HC had more access to APIP premises. This difference could result in patients having increased levels of tiredness secondary to increased travel time and stress before intervention.

The positive effects over quality of life are in accordance with previous findings (41) of positive correlation of the 6MWT with SF-36 scale, although high BMI in patients with SCZ could have reduced quality of life effects. The positive effect of walking activities, even indoor, such in the study, is supported by Hanson and Jones (24), with outdoor walks providing better health, quality of life, and physical functional capacity in normal subjects (42) and in patients with SCZ (25).

## Conclusion

The present study provided modest evidence of APIP over different outcomes in patients with SCZ (quality of life, psychopathology, and physical capacity), in contrast with similar programs that induced different effects in patients and HC even paired by age, sex, and social level. Additionally, it demonstrated that patients with SCZ have lower physical capacity and performance than HC in similar programs. Further studies can provide more benefit addressing additional items like personal lifetime developmental characteristics and can provide specific motivational interventions prior and during the program, promoting cessation or change in beta-blocker use, and provision of tailored equipment to patient's physical and mental handicaps. Although there is established evidence of benefits in PA, in the study, only a small percentage of patients with SCZ reached recommended levels of PA, even with standard APIP. These patients may have reduced improvement due to increased functional and motor impairment secondary to multiple factors such as physical inactivity, drug side effects, neurodevelopmental delays, and illness progression over body and brain. These factors must be considered in additional studies and interventions to help the patient come out of inactivity and to increase involvement in regular PA and better lifestyle.

## Data Availability Statement

All datasets generated for this study are included in the article/supplementary material.

## Ethics Statement

The studies involving human participants were reviewed and approved by Brazilian Research Registry (Plataforma Brasil) under number 43408615.7.0000.5327. The patients/participants provided their written informed consent to participate in this study.

## Author Contributions

MS participated in the elaboration of the research project, data collection, intervention, statistical analyses, and writing of the manuscript. VB collaborated in data collection. PB-A provided orientation in project elaboration, data acquisition, training in clinical scales, statistical analyses, and writing of the manuscript. All authors contributed to manuscript review and have read and approved the submitted version.

## Conflict of Interest

The authors declare that the research was conducted in the absence of any commercial or financial relationships that could be construed as a potential conflict of interest.
